# *Notes from the Field*: Ongoing Cholera Epidemic — Tanzania, 2015–2016

**DOI:** 10.15585/mmwr.mm6606a5

**Published:** 2017-02-17

**Authors:** Rupa Narra, Justin M. Maeda, Herilinda Temba, Janneth Mghamba, Ali Nyanga, Ashley L. Greiner, Muhammad Bakari, Karlyn D. Beer, Sae-Rom Chae, Kathryn G. Curran, Rachel B. Eidex, James J. Gibson, Thomas Handzel, Stephen J. Kiberiti, Rogath S. Kishimba, Haji Lukupulo, Theophil Malibiche, Khalid Massa, Amani E. Massay, Lindsey S. McCrickard, Geofrey J. Mchau, Vida Mmbaga, Ahmed A. Mohamed, Elibariki R. Mwakapeje, Emmanuel Nestory, Anna E. Newton, Elvis Oyugi, Anu Rajasingham, Michelle E. Roland, Neema Rusibamayila, Senga Sembuche, Loveness J. Urio, Tiffany A. Walker, Alice Wang, Robert E. Quick

**Affiliations:** ^1^Epidemic Intelligence Service, CDC; ^2^Division of Foodborne, Waterborne, and Environmental Diseases, National Center for Emerging and Zoonotic Infectious Diseases, CDC; ^3^Tanzanian Ministry of Health, Community Development, Gender, Elderly and Children, Dar es Salaam, Tanzania; ^4^Division of Global Health Protection, Center for Global Health, CDC; ^5^CDC Tanzania, Dar es Salaam, Tanzania; ^6^Tanzania Field Epidemiology and Laboratory Training Program, Dar es Salaam, Tanzania; ^7^Kenya Field Epidemiology and Laboratory Training Program, Nairobi, Kenya; ^8^Office of Noncommunicable Diseases, Injury and Environmental Health, National Center for Environmental Health, CDC.; * These authors contributed equally to this report.

On August 15, 2015, the Tanzanian Ministry of Health, Community Development, Gender, Elderly and Children (MOHCDGEC) was notified about a case of acute watery diarrhea with severe dehydration in a patient in Dar es Salaam. *Vibrio cholerae* O1, biotype El tor, serotype Ogawa, was isolated from the patient’s stool and an investigation was initiated. MOHCDGEC defined a suspected cholera case as the occurrence of severe dehydration or death from acute watery diarrhea in a person aged ≥5 years, or acute, profuse watery diarrhea with or without vomiting in a person aged ≥2 years in a region with an active cholera outbreak. A confirmed cholera case was defined as isolation of *V. cholerae* O1 from the stool of a person with suspected cholera. Tanzania’s first reported cholera epidemic was in 1974 with intermittent outbreaks since then; the largest epidemic occurred in 1997, with 40,249 cases and 2,231 deaths (case fatality rate [CFR] was 5.5%) (*1*).

As of November 26, 2016, the current epidemic continues, affecting 23 (92%) of 25 regions in mainland Tanzania (excluding the Zanzibar archipelago), with a cumulative reported case count of 23,258 and a cumulative CFR of 1.5%. The median number of reported cholera cases per week was 271 (range = 5–1,240) (Figure). Approximately half of all reported cases have been from four regions: Dar es Salaam (5,104; 22%), Morogoro (3,177; 14%), Mwanza (2,311; 10%), and Mara (2,299; 10%). Of 511 stool specimens tested during August 17, 2015–March 18, 2016 at the National Health Laboratory-Quality Assurance Training Center in Dar es Salaam, 268 (52%) were positive for *V. cholerae;* all specimens were serogroup O1, biotype El tor, serotype Ogawa. Antimicrobial resistance (AMR) testing revealed sensitivity to cotrimoxazole, ceftriaxone, tetracycline, ciprofloxacin, and chloramphenicol, and resistance to nalidixic acid and ampicillin.

**FIGURE:**
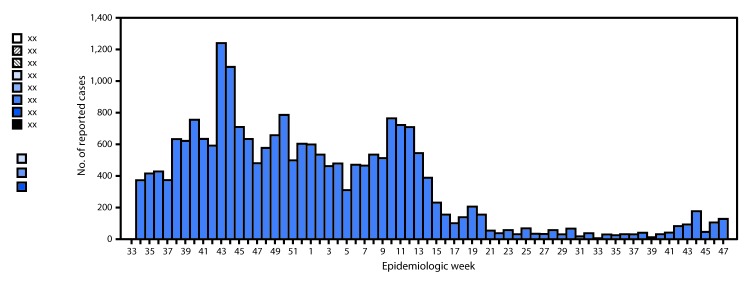
Number of reported cholera cases* — Tanzania,^†^ August 15, 2015–November 26, 2016 * Suspected cholera (severe dehydration or death from acute watery diarrhea in a person aged ≥5 years, or acute, profuse watery diarrhea with or without vomiting in a person aged ≥2 years in a region with an active cholera outbreak) and confirmed cases (isolation of *V. cholerae* O1 from the stool of a person with suspected cholera). ^†^ Excluding the Zanzibar archipelago.

The Tanzania Field Epidemiology and Laboratory Training Program and CDC in the United States and Tanzania evaluated cholera mortality reporting in Dar es Salaam; 81 deaths were identified during August 15–October 28, 2015. Cholera treatment center (CTC) records revealed that 21 (26%) patients died in CTCs. Municipal burial permits recorded 60 (74%) cholera deaths in the community. These results motivated follow-up interviews with decedents’ family members to identify characteristics associated with an increased risk for death from cholera in January 2016.

By October 2015, MOHCDGEC collaborated with partner organizations, including Médecins Sans Frontières, Tanzania Red Cross National Society, United Nations Children’s Emergency Fund, World Health Organization, and CDC, to enhance cholera response activities. To strengthen cholera surveillance, MOHCDGEC disseminated standardized cholera case definitions, developed reporting tools and communication strategies, produced daily situation reports, and distributed weekly summary reports to national and regional levels. Trainings on isolation, identification, and AMR testing of *V. cholerae* were conducted for regional and district laboratory staff members. MOHCDGEC used AMR test results to develop cholera treatment guidelines that emphasized appropriate antibiotic use in moderate to severe cases, and developed standard operating procedures for appropriate use of rapid diagnostic test kits for *V. cholerae* in regions at risk for cholera importation. A train-the-trainer approach was used to increase the pool of available responders to send to highly affected regions to strengthen cholera case management and infection prevention and control in health care facilities and CTCs. Partner organizations are mapping private water vendors in Dar es Salaam to facilitate chlorination of water tanks, encouraging municipal water authorities in Dar es Salaam and other cities to increase chlorine levels in piped water supplies, and targeting communities at high risk for distribution of household water treatment tablets (*2*). Cholera prevention and treatment materials have been developed and disseminated through mass media, health care facilities, CTCs, and door-to-door.

Tanzania continues to face challenges with epidemic control. Cholera outbreaks have been reported in most countries neighboring Tanzania, including Kenya, Uganda, the Democratic Republic of the Congo, Burundi, Malawi, Mozambique, and Zambia (*3*,*4*). In addition, the El Niño phenomenon, which has exacerbated cholera transmission in the past in the Great Lakes region of Africa (*5*), caused heavy rains throughout East Africa. To help address the challenges of this epidemic, MOHCDGEC established a national Emergency Operations Center in November 2015 and developed a National Cholera Response Plan in February 2016. The Emergency Operations Center is currently coordinating the deployment of multidisciplinary rapid response teams to support affected regions and districts. These activities have improved the ability of Tanzania to respond to this epidemic and serve as a model for responding to future public health emergencies.

## References

[1] World Health Organization Global Task Force on Cholera Control. Cholera country profile: United Republic of Tanzania, 2008. Geneva, Switzerland: World Health Organization; 2008. http://www.who.int/cholera/countries/TanzaniaCountryProfile2008.pdf

[2] Wang A, Hardy C, Rajasingham A, Notes from the field: chlorination strategies for drinking water during a cholera epidemic—Tanzania, 2016. MMWR Morb Mortal Wkly Rep 2016;65:1150–1. 10.15585/mmwr.mm6541a6 27764079

[3] CDC. Cholera—*Vibrio cholerae* infection: cholera in Africa. Atlanta, GA: US Department of Health and Human Services, CDC; 2016. https://www.cdc.gov/cholera/africa/index.html

[4] George G, Rotich J, Kigen H, Notes from the field: ongoing cholera outbreak—Kenya, 2014–2016. MMWR Morb Mortal Wkly Rep 2016;65:68–9. 10.15585/mmwr.mm6503a7 26820494

[5] Pascual M, Rodó X, Ellner SP, Colwell R, Bouma MJ. Cholera dynamics and El Niño southern-oscillation. Science 2000;289:1766–9. 10.1126/science.289.5485.1766 10976073

